# Adenomyosis-Associated Ischemic Stroke: Pathophysiology, Detection and Management

**DOI:** 10.3390/brainsci12101410

**Published:** 2022-10-20

**Authors:** Yuying Yan, Xuening Zhang, Di Zhong, Anmo Wang, Simiao Wu, Bo Wu

**Affiliations:** Department of Neurology, West China Hospital, Sichuan University, Chengdu 610041, China

**Keywords:** ischemic stroke, adenomyosis, thromboembolism, CA-125 antigen, menstruation

## Abstract

Female-specific risk factors for stroke have gradually received attention. The relationship between ischemic stroke and adenomyosis, a benign uterine disorder commonly present in parous women, is underrecognized. We aimed to provide an overview of the epidemiology, pathophysiological mechanisms, clinical characteristics, diagnostic considerations, and potential therapeutic strategies of adenomyosis-associated ischemic stroke. We shared our experience with the diagnosis and management of a patient, and summarized current findings and knowledge gaps of this disease based on previous literature. The relevant studies were searched in English and Chinese databases up to April 2022 using the keywords “ischemic stroke”, “cerebral infarction” and “adenomyosis”. Then, we provided a narrative review of the retrieved articles. Finally, the data of 32 cases were analyzed. We found that increased levels of carbohydrate antigen 125 and D-dimer and decreased level of hemoglobin are biomarkers of adenomyosis-associated ischemic stroke. In addition, hypercoagulability might be a key mechanism leading to thromboembolism in the cerebrovascular system. Additional studies are needed to find optimal prevention strategies for the disease. A better understanding of this “rare” pathogenesis of ischemic stroke may inform a more precise diagnosis and effective prevention strategy in middle-aged women with embolic stroke of undetermined source.

## 1. Introduction

Stroke is a leading cause of death and disability worldwide [1,2]. Largely attributed to their longer life expectancy, women face a greater lifetime stroke risk than men [3,4]. It is important to understand female-specific mechanisms of stroke and to optimize stroke management in women. Conventional vascular risk factors, such as hypertension, diabetes mellitus, hyperlipidemia, cigarette smoking, and atrial fibrillation, apply to both men and women and can partly explain the stroke incidence in women. With more attention being paid to diversity in stroke pathophysiology, female-specific risk factors for stroke, for example, endogenous hormones, exogenous estrogens, and pregnancy exposures, have gradually received attention in recent years [5,6].

Adenomyosis is a uterine disorder presenting with endometrial glands and stroma within the myometrium, resulting in hypertrophy of the surrounding myometrium, which is commonly diagnosed in parous women during their forties and fifties [7,8,9]. The primary symptoms of adenomyosis include abnormal uterine bleeding, pelvic pain, and infertility [7,8]. It is a benign gynecological disease that is a common condition in women. Its prevalence was reported to range from 0.8% to 70%, with an average of 20% to 35% [9,10,11]. Some patients with adenomyosis may develop ischemic stroke, but little is known about whether adenomyosis is a risk factor for ischemic stroke.

This study aimed to review previous literature combined with our experience and summarize the epidemiology, pathophysiological mechanisms, clinical characteristics, diagnostic considerations, and therapeutic strategies of adenomyosis-associated ischemic stroke. To the best of our knowledge, few reviews of adenomyosis-associated ischemic stroke have been published. The overview and summary of multiple aspects of the disease, rather than detailed information from specific cases, may provide a comprehensive understanding of this subtype of stroke. This review highlights adenomyosis as a possible cause of stroke. Recognizing this entity may help to make a precise diagnosis and implement effective prevention in female patients with cryptogenic stroke.

## 2. Our Experience and Methods of Literature Review

The authors’ experience with the diagnosis and management of a female patient with cryptogenic stroke possibly associated with adenomyosis revealed a “rare” and underrecognized cause of ischemic stroke. A 49-year-old Chinese woman had four ischemic strokes involving multiple vascular territories. Interestingly, three of the attacks occurred on the first day of her menstruation, which implied a possible gynecological mechanism of stroke. Her medical history and extensive examinations showed that she did not have conventional vascular risk factors but presented with elevated levels of carbohydrate antigen 125 (CA125) and D-dimer and moderate anemia (Table 1).

For further understanding, we conducted a comprehensive literature search concerning adenomyosis-associated ischemic stroke in English databases (PubMed, Embase and Web of Science) and Chinese databases (WANFANG database and China Knowledge Resource Integrated Database [CNKI]). The keywords “ischemic stroke”, “cerebral infarction” and “adenomyosis” were used to identify relevant articles. We searched all types of studies published in English and Chinese from January 1992 to April 2022. Two independent reviewers screened the titles and abstracts to assess the eligibility of articles after the database search. Then, the relevant information was extracted from the retrieved studies. The search strategy is summarized in Table 2.

The first report about adenomyosis-associated ischemic stroke was published in 2012, which described a 42-year-old woman who developed cerebral infarction with a 10-year history of adenomyosis [12]. In the following decade, only scattered cases or case series have been reported on the topic (Table 1) [13,14,15,16,17,18,19,20,21,22,23,24,25,26,27]. This review analyzed the features of 32 cases.

## 3. Epidemiology of Adenomyosis-Associated Ischemic Stroke

Accumulating evidence suggests that cancer may increase the risk of stroke [28]. Approximately 10% of hospitalized patients with ischemic stroke in the United States have cancer [29]. In addition, 10% to 20% of patients with stroke of undetermined cause have an occult malignancy [30,31]. The association between ischemic stroke and malignant tumors has been well recognized [32,33]. However, little is known about this relationship in patients with adenomyosis, a benign gynecologic tumor.

The true incidence and prevalence of adenomyosis-associated ischemic stroke remain uncertain. It can be inferred that the rate of stroke due to a benign tumor is much lower than that of malignancy-related stroke. One case series identified four patients with adenomyosis (0.8%) among 521 patients with cerebral infarcts [13]. Similarly, one patient with embolic stroke considered to be related to adenomyosis (0.1%) was recognized in an ischemic stroke cohort of 709 patients [14]. Another study retrospectively analyzed 5570 patients with adenomyosis and identified 11 patients (0.2%) with ischemic stroke [22].

## 4. Pathophysiology of Adenomyosis-Associated Ischemic Stroke

### 4.1. Mechanisms of Hypercoagulability

Adenomyosis patients are at risk of having a dysfunctional coagulation system and thrombotic disorders [16,34]. Several speculative mechanisms of activated coagulation are discussed here. Higher levels of procoagulant factors, such as tissue factor (TF) and mucinous protein, potentially increase the risk of thrombus formation.

Adenomyosis and endometriosis share remarkable similarities in definition, pathophysiology, and molecular aberrations [7]. TF expression is elevated in eutopic and ectopic endometria in endometriosis patients [35]. A previous study suggested that TF immunoreactivity in the endometrium was significantly increased in women with adenomyosis-associated heavy menstrual bleeding and dysmenorrhea [36]. TF released into the circulation plays a pivotal role in the activation of the clotting cascade [37]. Additional studies are needed to support this hypothesis.

Mucins are large and heavily glycosylated molecules secreted by epithelial cells that promote thrombosis through bidirectional signaling in platelets and neutrophils, ultimately generating platelet-rich microthrombi [38,39]. In addition to adenocarcinoma, elevated serum levels of CA125, a typical mucin molecule, are also detected during menstruation and pregnancy and in patients with endometriosis [40].

Furthermore, rodent models of adenomyosis revealed that activated platelets were vital in the pathophysiological process of adenomyosis [41]. Anti-platelet treatment was effective in the suppression of adenomyosis progression [42]. A retrospective study demonstrated that the mean platelet volume in patients with pathologically confirmed adenomyosis was larger than that in healthy controls but there was no difference in platelet counts [43]. Larger platelets can express and release more thromboxane A2 and are functionally more reactive, correlating with aggregation during thrombotic events [44].

### 4.2. Embolic Sources

A hypercoagulable state is associated with a higher risk of ischemic stroke. Systemic thromboembolic events, in addition to cerebral infarction, might be discovered in this situation, such as finger or renal infarction and pulmonary embolism [13,25]. There are several possible embolic sources.

First, nonbacterial thrombotic endocarditis, as a disease that forms a sterile vegetation composed of fibrin and platelets on cardiac valves, commonly associated with malignancy and some autoimmune diseases [45], has been found to be the embolic source in some adenomyosis-associated stroke cases [15,17,18]. Second, the circulating mucin emboli might directly lead to cerebral infarction when the CA125 concentration is considered extremely high (>480 U/mL) [46]. Third, deep venous thrombosis, including pulmonary embolism and the lower limb deep-vein thrombosis observed in some patients with adenomyosis, are speculated to cause stroke via paradoxical embolization [14,47]. However, further autopsy data or pathological analysis of the emboli specimen are needed to ascertain these hypotheses of thrombus formation.

### 4.3. Triggering Factors

There are some other factors that may trigger ischemic stroke in patients with adenomyosis. 

Inflammation is a possible trigger. Six cases, including the one in the authors’ center, were presented with fever [17,21,23,24,25]. Some targets of the immune system are shared with the coagulation system, therefore acute infection may initiate the inflammatory response and induce a hypercoagulable state. Furthermore, chronic inflammation has a cumulative effect of vascular injury, resulting in premature atherosclerosis [48]. It is supposed that with an acute or chronic infection, inflammation in the uterus or other organs may be associated with a higher risk of ischemic stroke.

In addition, the overlap between the menstruation duration and the onset of stroke in some cases implies that stroke risk may increase during this period. Although rarely reported, menstruation might cause coagulopathy (i.e., disseminated intravascular coagulation) [13]. Heavy menstrual bleeding, one of the typical presentations of adenomyosis, could lead to anemia, a hyperkinetic state that disturbs endothelial adhesion molecule genes and may lead to thrombus formation. Augmentation and turbulence of blood flow may further result in embolism by migrating the thrombus [49].

Moreover, hormone therapy might be related to cerebrovascular disease. Combined oral contraceptives, comprised of estrogen and progestogen, are the commonly used medical treatment for adenomyosis [50]. It is noted that oral estrogens have a dose-response association with ischemic stroke risk [51]. It has also been reported that long-term hormone replacement therapy could aggravate adenomyosis and lead to ischemic stroke [15].

**Figure 1:**
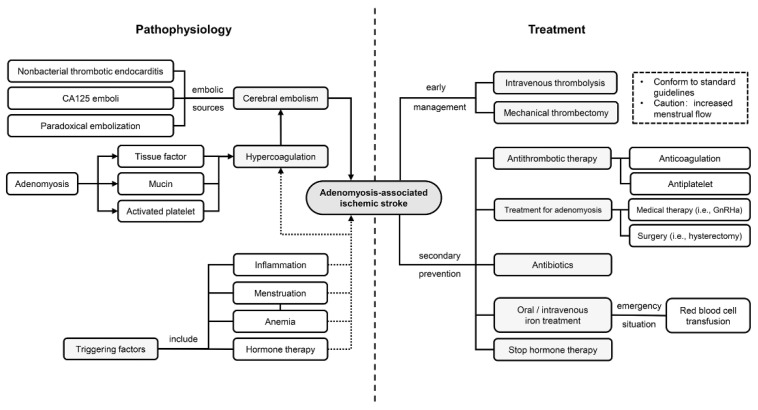
Underlying pathophysiology and treatment strategies in adenomyosis-associated ischemic stroke. A hypercoagulation state, potentially caused by excessive tissue factor and mucinous protein and activated platelets, leads to cerebral embolism. Several factors, such as menstruation and inflammation, may trigger the pathophysiological process. Management strategies mainly consist of antithrombotic therapy and treatments targeting adenomyosis. Abbreviation: CA125: carbohydrate antigen 125; GnRHa: gonadotropin releasing hormone analogs.

Figure 1 illustrates the hypothesized mechanism of ischemic stroke in patients with adenomyosis.

## 5. Clinical Characteristics and Diagnosis of Adenomyosis-Associated Ischemic Stroke

### 5.1. General Information

Patients with adenomyosis-associated ischemic stroke have been reported in Japan, China and Korea [13,14,15,16,17,18,19,20,21,22,23,24,25,26,27]. It is commonly diagnosed in middle-aged women, with the patients’ ages in the literature ranging from 34 to 59 years. Adenomyosis is an estrogen-dependent disease, rarely diagnosed in premenarchal or postmenopausal women [52], indicating the same tendency in ischemic stroke with adenomyosis. Medical history and examinations of cases suggest that most of the patients have no or, if any, only a couple of conventional vascular risk factors, such as smoking, hypertension, diabetes, or heart disease.

### 5.2. Clinical Manifestation

Although conflicting data exist, most studies have indicated that multiple and minor strokes in both cortical and subcortical areas tend to be the major stroke type in patients with adenomyosis-associated ischemic stroke, presenting with different degrees of focal neurological deficits, such as dysarthria, hemiparesis, and sensory disturbance. Occlusion of the proximal segment of the intracranial arteries was observed in some cases [17,19,21], resulting in severe neurological deficits.

Ischemic stroke occurring during the menstrual phase with or without fatigue and dizziness could be a unique characteristic and a pivotal clue for diagnosis [13,20,21,22,23,24,25,26,27]. Dysmenorrheal and heavy menstrual bleeding are two of the common adenomyosis symptoms, also occurring in nearly half of the cases with adenomyosis-associated ischemic stroke. However, it is recognized that some patients are asymptomatic with abnormal uteri.

Moreover, an elevated temperature indicating probable infection could manifest at an early stage or even before the disease [17,21,23,24,25]. It is possible that ischemic stroke occurs with systemic thromboembolism involving multiple organs or arteries, such as renal infarction and pulmonary embolization [13,21,25,26]. Six cases including ours experienced recurrent stroke and neurological deterioration [12,21,25,26,27], most of which had a close relationship with irregular menstrual bleeding.

### 5.3. Laboratory Tests

For laboratory analysis, increased levels of CA125 and D-dimer and a reduction in hemoglobin concentration are the three principal biomarkers in ischemic stroke patients with adenomyosis. Other biomarkers include carbohydrate antigen 19-9 (CA19-9) and inflammatory markers (i.e., C-reactive protein) [14,17,20,21,23,25,26,27]. 

A serum concentration of CA125 over 35 U/mL is considered as abnormal, while its fluctuation is physiologic within a normal menstrual cycle (the mean peak level may reach 51.8 U/mL during menstruation) [53]. CA125 in patients with adenomyosis-associated ischemic stroke often exceeds the physiologic fluctuation extent, reaching several-fold higher than the upper limit of the normal range. The highest one reported in previous cases even rose to 4276.7 U/mL.

D-dimer is a nonspecific biomarker of hypercoagulability. A study compared D-dimer levels among eight adenomyosis patients and found that they were only increased in the patients with cerebral infarction or pulmonary thromboembolism and/or hemoglobin loss >20 g/L during menstruation [16].

These parameters might also serve as surrogate markers for monitoring the response to therapy in patients with adenomyosis-associated stroke. The values of CA125, CA19-9 and D-dimer decreased to lower levels or returned to normal ranges after treatment within weeks or months [12,15,20,21,23,24,25,26,27].

### 5.4. Neuroimaging

Approximately two-thirds (68.8%) of the cases demonstrated embolic-appearing infarcts in the bilateral anterior and posterior circulations or were disseminated in at least two artery territories. Intracranial and extracranial vessels should be carefully evaluated. Vessel-wall magnetic resonance imaging (MRI) and magnetic resonance venography could be performed, if possible, to exclude certain vasculopathies (i.e., atherosclerosis, arterial dissection, or vasculitis) and venous sinus thrombosis.

### 5.5. Adenomyosis

Histopathological examination is still the strictest criterion of adenomyosis diagnosis, and it eliminates the possibility of uterine malignancy considering the abnormal CA125 level. Some noninvasive techniques such as sonography and MRI are effective methods to assist in diagnosis [54]. A systematic review suggested that MRI is more accurate in diagnosis than ultrasound [55]. Transvaginal or transabdominal ultrasonography is regarded as a first-line test to detect adenomyosis considering its advantage of a lower cost, while MRI is a complementary examination after a nonconclusive ultrasonography evaluation [54].

However, not all patients with adenomyosis are at risk of ischemic stroke. A study demonstrated that adenomyosis patients with uterine volume over 100 cm^3^, multiple hyperintensive spots on T2-weighted MRI inside the adenomyosis tissues and elevated soluble fibrin, and D-dimer during menstruation might have a higher risk of thrombotic disorders, possibly due to an activated coagulation system [16]. Another study compared the CA125 levels between groups of different enlargement degrees of the uterus with or without adenomyosis, indicating that severe adenomyosis, particularly with a uterine volume over 240–300 cm^3^ or size greater than 12 weeks, could cause an abnormal CA125 rise [56].

### 5.6. Other Examinations for Exclusive Diagnosis

The diagnosis of adenomyosis-associated ischemic stroke is a process of exclusion of many other potential etiologies.

A few evaluations are imperative for the diagnosis of this possible subtype of cryptogenic stroke. Transesophageal echocardiography with agitated saline injection after transthoracic echocardiography is recommended to detect any cardiogenic risk factor, such as patent foramen ovale, aortic atherosclerosis, and nonbacterial thrombotic endocarditis [57]. Prolonged cardiac rhythm monitoring is useful for detecting paroxysmal atrial fibrillation [58]. A proportion of unexplained stroke caused by inherited and acquired thrombophilia may be excluded after checking a list of conditions, including anti-thrombin III deficiency, prothrombin gene mutation, Factor V Leiden mutation, protein C and S deficiency, antiphospholipid antibody syndrome, methylenetetrahydrofola reductase mutation, and hyperhomocysteinemia [59]. Cerebrospinal fluid analysis might be required due to a suspicion of infection or vasculopathies. Cancer-associated stroke is another important differential diagnosis as it shares some characteristics with adenomyosis, for example, elevated tumor biomarkers of CA125 and CA19-9 [32]. Noninvasive imaging approaches, including chest, abdomen and pelvic enhanced imaging, are effective for screening occult malignancies from the lung, pancreas, and ovaries, and evaluating whether any systemic thromboembolism involves other organs.

**Figure 2:**
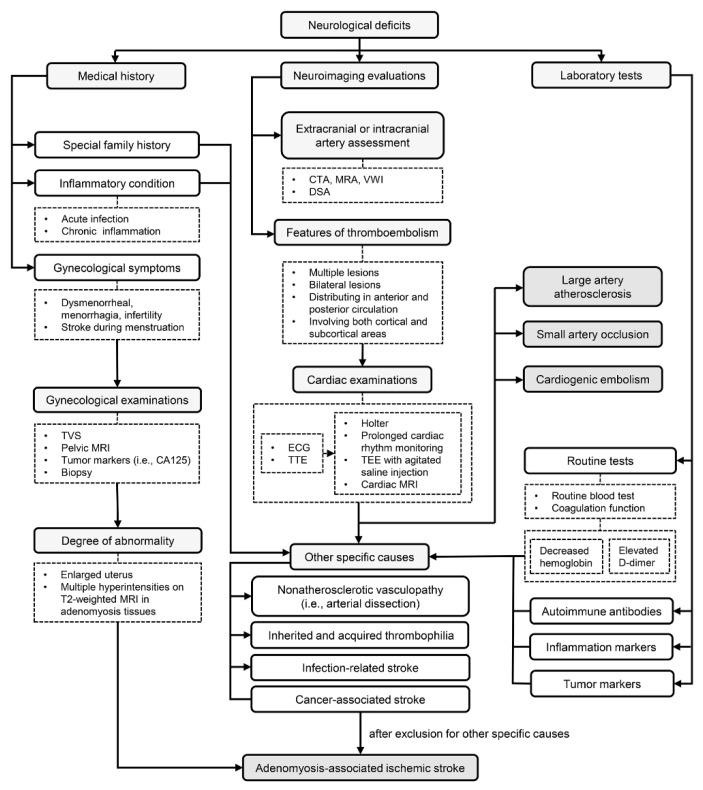
A diagnostic flow chart of adenomyosis-associated ischemic stroke. Medical history of gynecological symptoms, neuroimaging features of thromboembolism and laboratory test results of increased D-dimer, elevated CA125 and decreased hemoglobin are pivotal clues in disease recognition. Large artery atherosclerosis, small artery occlusion, cardiogenic embolism and some other specific causes should be carefully excluded. Abbreviation: TVS: transvaginal ultrasonography; MRI: magnetic resonance imaging; CA125: carbohydrate antigen 125; CTA: computerized tomography angiography; MRA: magnetic resonance angiography; VWI: vessel wall imaging; DSA: digital subtraction angiography; ECG: electrocardiograph; TTE: transthoracic echocardiography; TEE: transesophageal echocardiography.

Based on existing evidence and experience in practice, we proposed a diagnostic procedure, as shown in Figure 2.

## 6. Treatment Considerations for Adenomyosis-Associated Ischemic Stroke

### 6.1. Early Management

As a benign tumor and a common condition among women, adenomyosis is predisposed to be underrecognized during the early stage of an acute ischemic stroke attack. The application of recanalization and reperfusion treatment for stroke patients, including intravenous thrombolysis and mechanical thrombectomy, should comply with clinical guidelines in which adenomyosis is not seen as an absolute contraindication [60].

However, menstruation during ischemic stroke as active bleeding condition is a theoretical contraindication to intravenous tissue plasminogen activator (tPA) treatment. A review of cases indicated that intravenous tPA might be used relatively safely in women who are menstruating but cautioned against the administration of tPA to patients with a history of dysfunctional uterine bleeding. These patients may have a transfusion requirement due to increased menstrual flow [61].

No comparison has been made between the effectiveness and safety of recanalization and conservative treatment for adenomyosis-associated ischemic stroke.

### 6.2. Antithrombotic Therapy

Antithrombotic agents are the primary strategy for the secondary prevention of ischemic stroke. Studies concerned with whether anticoagulants or antiplatelets are the optimal strategy for treating adenomyosis-associated ischemic stroke are limited.

Anticoagulation therapy is presumed to be the best option and it is often empirically performed in practice based on the strong theoretical consideration of a hypercoagulable status as the primary mechanism. Heparin-bridging anticoagulation therapy was the most widely used method in previous cases. One patient changed from antiplatelet to anticoagulation therapy due to recurrent stroke approximately 14 months after the first attack [12]. Conversely, our patient was changed from factor Xa inhibitor to antiplatelet therapy because of anemia caused by increased menstrual flow. Unfortunately, anemia and menorrhagia might be related to stroke recurrence. Although anticoagulant therapy may reduce the risk of recurrent thromboembolism, it should be carefully weighed against the increased risk of uterus bleeding.

### 6.3. Treatment for Adenomyosis

In addition to antithrombotic treatment, therapies targeting adenomyosis, which is directly linked to the pathogenesis of this stroke subtype, are also important. There are no specific guidelines to follow for the management of adenomyosis.

Currently, medical therapy, including progestins, combined oral contraceptives, gonadotropin releasing hormone (GnRH) analogs, and levonorgestrel-releasing intrauterine systems, have shown increased efficacy in patients requiring control of symptoms or fertility treatments [50]. GnRH analogs were the most commonly used hormonal treatment drug in previous cases. They can effectively ameliorate heavy menstrual bleeding and dysmenorrhea and reduce uterine size, but they also have hypoestrogenic side effects [9]. Combined oral contraceptives should be carefully prescribed due to the increased risk of arterial thrombotic events, particularly among those who have other vascular risk factors [6].

Although indications for hysterectomy and other surgical treatments differ depending on the surgeon, this is a choice for patients whose dysmenorrhea and hypermenorrhea are difficult to control with medication and who have no desire for future fertility [62,63]. Several patients still suffered recurrent strokes after receiving anticoagulation with pseudomenopause treatment, but successfully prevented infarctions after adenomyosis resection, indicating that hysterectomy may be an effective and radical therapy in these cases [21,27].

Some laboratory examinations, such as CA125, CA19-9, D-dimer and hemoglobin concentrations, are promising biomarkers to monitor the treatment effect and disease prognosis.

### 6.4. Other Treatments

Common complications of the disease should be appropriately addressed. Iron deficiency anemia is commonly reported in women with heavy menstrual bleeding, who can be provided with oral and intravenous iron treatment, and in emergency situations, red blood cell transfusion is the main replacement option [64]. Infection, if any, should be effectively controlled by antibiotics to prevent deterioration due to acute inflammation. Figure 1 summarizes the potential treatment strategies for patients with adenomyosis-associated ischemic stroke.

## 7. Future Directions

The optimal long-term management strategy for adenomyosis-associated ischemic stroke remains uncertain. Anticoagulation may benefit these patients if an increased risk of bleeding can be avoided. Hysterectomy might be an alternative treatment in patients without fertility desires. Advances in artificial intelligence-based medicine might be promising to formulate a diagnosis, suggest therapeutic options and estimate the prognosis in a more individualized way for female patients with embolic stroke of undetermined source.

There is an urgent need for research focused on a series of problems. The first is whether it is necessary for patients with adenomyosis at a high risk of stroke (i.e., increased CA125 and D-dimer) to take primary prevention measures. Second, a time point to reassess or stop the antithrombotic treatment is unknown. Third, for patients with adenomyosis-associated ischemic stroke of child-bearing age, it is unclear how to evaluate the risk of stroke recurrence after pregnancy.

## 8. Conclusions

Adenomyosis is a common benign tumor but an underrecognized risk factor for ischemic stroke, which may threaten the health of middle-aged women. The hypercoagulable state induced by adenomyosis-derived mucin might be the key mechanism of thromboembolism in adenomyosis-associated ischemic stroke. Typical characteristics include elevated levels of serum CA125 and D-dimer as well as heavy menstruation-related anemia. The fluctuations of these parameters are also predictors of stroke recurrence and the treatment effect.

In conclusion, clinicians should be aware of adenomyosis as a female-specific risk factor for thromboembolism, which may lead to ischemic stroke. Comprehensive management regarding both aspects of neurology and gynecology can successfully prevent from stroke occurrence.

## Figures and Tables

**Table 1:** Reported cases of adenomyosis-associated ischemic stroke.

					Laboratory Test			Therapy			
Patient Number	Age	Occurrence during Menstruation	Systemic Embolism	NTBE	D-Dimer (μg/mL; Normal, <0.5 μg/mL)	CA125 (U/mL; Normal, <35 U/mL)	Hemoglobin (g/L; Normal, 110–150 g/L)	Anti-Thrombotic Treatment	Treatment for Adenomyosis	Recurrence	Reference Number
1	42	Yes	/	No	6.0	1750	86	Antiplatelet therapy	GnRH agonist	Yes	[12,13]
	43	Menstrual phase started 7 days before admission	/	No	4.1	907	/	Heparin, warfarin	GnRH agonist	No (4 m)	
2	45	No	Yes	No	1.1	159	84	Heparin, antiplatelet therapy	GnRH agonist	/	[13]
3	44	/	Yes	No	/	/	70	Heparin, warfarin	GnRH agonist	/	
4	50	Yes	/	No	0.57	42.6	69	Aspirin	GnRH agonist	/	
5	57	/	/	No	11.16	1470	/	/	/	/	[14]
6	59	/	/	Yes	7.0	334.8	/	Heparin	Stopped HRT	No (6 m)	[15]
7 ^†^	38	/	/	/	Increased	/	Loss 27 g/L during menstruation	/	/	/	[16]
8	48	/	/	Yes	1.9	901	85	Heparin, warfarin	Hysterectomy	No (6 m)	[17]
9	49	Menorrhagia happened 10 days before admission	No	Yes	3.99	379	99	Enoxaparin, vitamin K antagonist	Hysterectomy	No (4 m)	[18]
10	42	/	No	No	1.4	395	/	Warfarin	/	No (68 m)	[19]
11	50	/	No	No	3.7	143	/	Rivaroxaban	/	No (19 m)	
12	34	Yes	/	No	1.05	937.1	134	/	/	/	[20]
13	37	Yes	/	No	2.34	735.7	108	/	/	/	
14	46	Yes	/	No	12.04	546.5	121	/	Hysterectomy	No	
15	44	Yes	Yes	No	17.0	2115	103	Heparin, rivaroxaban	/	Yes	[21]
	44	Yes	/	/	2.4	561	/	Warfarin	GnRH agonist	Yes	
	45	Irregular menstrual bleeding	/	/	22.0	1291.6	/	Warfarin	Hysterectomy	No (24 m)	
16–26 ^‡^	/	18.2% during menstruation	/	/	72.7% increased	90.9% increased	100% anemia	Antiplatelet therapy	9.1% GnRH agonist, 9.1% levonorgestrel, 18.2% GnRH agonist and levonorgestrel	/	[22]
27	34	Yes	/	No	27.4	937.7	112	Heparin, clopidogrel	/	No (4 m)	[23]
28	51	Yes	No	No	/	4276.7	123	Heparin, apixaban	GnRH agonist	No (6 m)	[24]
29	48	Yes	Yes	/	79.3	/	82	Heparin	/	Yes	[25]
	48	Heavy uterine bleeding	/	No	/	3536.2	/	Edoxaban	Hysterectomy	No (15 m)	
30	47	Yes	/	No	3.8	90.3	113	Heparin, edoxaban	/	Yes	[26]
	47	Yes	Yes	/	4.2	/	/	Heparin	Hysterectomy	No (60 m)	
31	50	Yes	/	No	6.4	999	92	Heparin, apixaban	GnRH agonist	Yes	[27]
	50	No	/	/	/	/	/	Heparin	Hysterectomy	No (18 m)	
32	44	Yes	/	/	/	/	/	Clopidogrel	/	Yes	The present case
	46	Yes	No	No	4.71	197	77	Rivaroxaban (stopped because of the bleeding and anemia), clopidogrel	Irregular treatment	Yes	
	48	Yes	No	No	8.64	341	72	Clopidogrel	GnRH agonist	Yes	
	49	No	/	No	2.16	12.9	131	Clopidogrel	GnRH agonist	No (15 m)	

^†^ Laboratory test data were obtained during menstrual phase. ^‡^ A case series consists of 11 patients. Slash indicates not mentioned. Abbreviation: NTBE: nonbacterial thrombotic endocarditis; CA125: carbohydrate antigen 125; GnRH: gonadotropin releasing hormone; HRT: hormone replacement therapy.

**Table 2:** Summary of search strategy.

Items	Specification
Databases and other sources searched	English databases (PubMed, Embase and Web of Science) and Chinese databases (WANFANG and CNKI)
Search terms used	“ischemic stroke”, “cerebral infarction”, “adenomyosis”
Timeframe	January 1992 to April 2022
Inclusion and exclusion criteria	All study types published in English and Chinese were collected.
Selection process	Two reviewers (Y.Y., X.Z.) conducted the selection independently. Relevant information was extracted by the other two reviewers (D.Z., A.W.). All the authors jointly discussed to obtain the consensus if there was any discrepancy.

Abbreviation: CNKI, China Knowledge Resource Integrated Database.

## Data Availability

The data presented in this study are available from the corresponding author upon reasonable request.
